# A new method to detect loss of heterozygosity using cohort heterozygosity comparisons

**DOI:** 10.1186/1471-2407-10-195

**Published:** 2010-05-12

**Authors:** Michael R Green, Paul Jardine, Peter Wood, Jeremy Wellwood, Rod A Lea, Paula Marlton, Lyn R Griffiths

**Affiliations:** 1 Genomics Research Centre, Griffith Institute for Health & Medical Research, Griffith University, Parklands Drive, Southport, Queensland, Australia; 2 Griffith Medical Research College, a joint program of Griffith University and the Queensland Institute of Health and Medical Research, QIMR, Herston Road, Herston, Queensland, Australia; 3 Research Computing Services, Griffith University, Parklands Drive, Southport, Queensland, Australia; 4 The Prince Charles Hospital, Queensland Health, Rode Road, Chermside, Queensland, Australia; 5 Gold Coast Hospital, Queensland Health, Nerang Road, Southport, Queensland, Australia; 6 Institute of Environmental Science and Research, Kenepuru Drive, Porirua, New Zealand; 7 Princess Alexandra Hospital, Queensland Health, Ipswitch Road, Woloongabba, Queensland, Australia

## Abstract

**Background:**

Loss of heterozygosity (LOH) is an important marker for one of the 'two-hits' required for tumor suppressor gene inactivation. Traditional methods for mapping LOH regions require the comparison of both tumor and patient-matched normal DNA samples. However, for many archival samples, patient-matched normal DNA is not available leading to the under-utilization of this important resource in LOH studies. Here we describe a new method for LOH analysis that relies on the genome-wide comparison of heterozygosity of single nucleotide polymorphisms (SNPs) between cohorts of cases and un-matched healthy control samples. Regions of LOH are defined by consistent decreases in heterozygosity across a genetic region in the case cohort compared to the control cohort.

**Methods:**

DNA was collected from 20 Follicular Lymphoma (FL) tumor samples, 20 Diffuse Large B-cell Lymphoma (DLBCL) tumor samples, neoplastic B-cells of 10 B-cell Chronic Lymphocytic Leukemia (B-CLL) patients and Buccal cell samples matched to 4 of these B-CLL patients. The cohort heterozygosity comparison method was developed and validated using LOH derived in a small cohort of B-CLL by traditional comparisons of tumor and normal DNA samples, and compared to the only alternative method for LOH analysis without patient matched controls. LOH candidate regions were then generated for enlarged cohorts of B-CLL, FL and DLBCL samples using our cohort heterozygosity comparison method in order to evaluate potential LOH candidate regions in these non-Hodgkin's lymphoma tumor subtypes.

**Results:**

Using a small cohort of B-CLL samples with patient-matched normal DNA we have validated the utility of this method and shown that it displays more accuracy and sensitivity in detecting LOH candidate regions compared to the only alternative method, the Hidden Markov Model (HMM) method. Subsequently, using B-CLL, FL and DLBCL tumor samples we have utilised cohort heterozygosity comparisons to localise LOH candidate regions in these subtypes of non-Hodgkin's lymphoma. Detected LOH regions included both previously described regions of LOH as well as novel genomic candidate regions.

**Conclusions:**

We have proven the efficacy of the use of cohort heterozygosity comparisons for genome-wide mapping of LOH and shown it to be in many ways superior to the HMM method. Additionally, the use of this method to analyse SNP microarray data from 3 common forms of non-Hodgkin's lymphoma yielded interesting tumor suppressor gene candidates, including the ETV3 gene that was highlighted in both B-CLL and FL.

## Background

The elimination of tumor suppressor gene (TSG) function contributes to carcinogenesis and cancer progression. Early work on the RB1 gene locus suggested two hits in TSGs were required to disrupt TSG function [1,2]. That is, both alleles of a TSG must be interrupted by mutation or allelic loss in order to render it inactive. Loss of heterozygosity (LOH), the transition from germ-line heterozygosity at a polymorphic locus to somatic homozygosity, is a hallmark of allelic loss and thus represents one of the two hits required for TSG inactivation.

Analysis of LOH is therefore important in cancer research in order to localize potential TSGs that may have a role in disease genesis and progression [3]. The introduction of high-density single nucleotide polymorphism (SNP) arrays has allowed high-resolution mapping of LOH and the delineation of minimally lost regions that indicate the presence of important TSGs [4]. The conventional method for LOH analysis relies on the comparison of SNP genotypes between tumor DNA samples and patient-matched control (germ-line) DNA samples obtained from normal (non-tumor) tissue. LOH determined in this manner will henceforth be referred to as 'conventional LOH'. The limitation of conventional methods of LOH analysis is that many archived tumor samples are not accompanied by patient-matched control tissue resulting in the under-utilization of these potentially valuable resources for interrogation of LOH.

Analysis of allelic copy number, using Hidden Markov Model (HMM)-based approaches such as QuantiSNP [5] and PennCNV [6], allow detection of LOH resulting from hemizygous deletion of single alleles, but lack the capacity to detect copy-number neutral LOH. Recently, an alternative method was described that utilizes a HMM to infer the presence of LOH from SNP microarray data of non-matched tumor samples by the absence of heterozygosity [7], thus allowing detection of copy-number neutral LOH. This method utilizes long strings of SNPs with homozygous calls to infer LOH and generates high numbers of candidate regions spanning large genetic distances. In order to overcome this limitation and increase the resolution of LOH mapping, we have developed a method to infer the presence of LOH regions through the comparison of SNP heterozygosity values between case and reference cohorts (Cohort Heterozygosity Comparison; CHC). The CHC method infers LOH candidate regions by the presence of short strings of SNPs which exhibit consistently lower prevalence of heterozygosity in the case cohort compared to the control cohort. The method is based on the hypothesis that decreased heterozygosity of closely spaced SNPs in the case cohort compared to the control cohort indicates a region of LOH. The CHC method calculates heterozygosity values for SNPs covering the entire genome in case and control cohorts, and then identifies and recovers the data from informative SNPs. By directly comparing heterozygosity of informative SNPs, groups of SNPs with significant differences in heterozygosity between the cohorts can be identified.

In this study, the CHC method was employed using SNP data from Affymetrix 250 K SNP arrays to infer LOH regions in tumor samples from B-cell Chronic Lymphocytic Leukemia (B-CLL), Diffuse Large B-cell Lymphoma (DLBCL) and Follicular Lymphoma (FL) patients. SNP array data from patient-matched buccal samples from four of the B-CLL patients was initially used to definitively map LOH by conventional methods in order to determine regions that will henceforth be referred to as 'conventional LOH'. These 'conventional LOH' regions were used in order to validate 'inferred LOH' regions generated by the CHC method. Validation of 'conventional LOH' was additionally performed by high-density microsatellite analysis. This work, as well as direct comparison of the efficacy of the CHC method with that of the only alternative (HMM) method, highlighted the utility of cohort heterozygosity comparisons for mapping LOH candidate regions without the need for patient-matched control samples. We therefore performed CHC analysis on larger numbers of FL, DLBCL and B-CLL samples in order to elucidate LOH candidate regions with potential roles in disease pathogenesis.

## Methods

### Patient Samples and DNA Extraction

Peripheral blood samples were obtained from B-CLL patients (n = 10) through the Prince Charles and Gold Coast Hospitals. Lymphocytes were enriched from 3 mL of whole-blood using ACCUSPIN™ system HISTOPAQUE^®^-1077 columns (Sigma-Aldrich) according to the manufacturer's protocol and B-cells were isolated from lymphocyte enrichments using Dynabeads^® ^CD19 (Invitrogen) and a magnetic particle concentrator according to the manufacturer's protocol. Following isolation of B-cells, the cells were lysed by the addition of 600 *μ*L of lysis buffer (Qiagen) and passing through a 20-guage needle. DNA was purified from lysed cells using QIAamp DNA Tissue Mini Kits according to the manufacturer's protocol. Buccal cell samples were collected from 6 of the B-CLL patients using Catch-All sample collection swabs (Epicentre Biotechnologies), and DNA isolated using BuccalAmp DNA extraction kits (Epicentre Biotechnology) according to the manufacturer's protocol.

FL (n = 20) and DLBCL (n = 20) tumor specimens were obtained through the Australian Leukemia and Lymphoma Group (ALLG) Tissue Bank (Princess Alexandra Hospital, Queensland, Australia) and BioOptions BioRepository Service http://www.biooptions.com/. DNA was extracted from tumor tissue using a modified column extraction protocol. 10 mg of tissue was homogenized in 1 mL of phosphate-buffered saline (pH 7.4) using a rotor-strator homogenizer. Protein was digested by incubation with Proteinase-K at 70°C for 30 min. DNA was purified from the milieu using QIAamp DNA Blood Midi Kit (Qiagen) according to the manufacturers protocol and all samples that were below a concentration of 100 ng/*μ*L were precipitated in ethanol and re-eluted in a smaller volume.

### Single Nucleotide Polymorphism Microarrays

DNA samples were amplified, fragmented, labelled and hybridized to Affymetrix 250 K Sty SNP microarrays in accordance with the manufacturer's protocol. Raw data was extracted from image files using GeneChip Operating System software (GCOS; Affymetrix). SNP genotyping and HMM-based LOH analysis was performed using GeneChip Genotyping Software (GTYPE; Affymetrix). It should be noted that the HMM-method does not utilize comparison to any normal sample, and generates LOH candidate regions based upon the genotypes generated from the tumour samples only. 'Conventional LOH' was detected in 4 B-CLL patients by comparison of SNP microarray genotypes from Buccal and tumor samples. This was performed using dChipSNP software as previously described [8].

### Microsatellite Analysis of LOH

Case tumor and buccal DNA samples were amplified using the Illustra Genomiphi V2 DNA amplification kit (GE Healthcare) according to the manufacturer's protocol. Each sample was genotyped at the Australian Genome Research Facility (AGRF, Sydney, Australia) using a standardized set of 63 microsatellites markers for chromosome 1. These markers span chromosome 1 with an average resolution of 5 cM http://appliedbiosystems.com/.

### Evaluation and Statistical Analysis of 'Inferred LOH' Regions

In order to evaluate the 'Inferred LOH' regions generated by CHC and HMM methods, annotation data from the Affymetrix GeneChip^® ^Genotyping Software was analyzed in Microsoft Excel. Each inferred LOH region was then plotted on the spreadsheet and evaluated for size and distance from SNPs showing 'conventional LOH'. Size was calculated using the physical position of the SNPs at the end of each region. The distance from 'conventional LOH' was calculated using the physical positions of the SNPs at the end of each region and the physical position of the closest SNP showing 'conventional LOH'. If candidate regions encompassed SNPs showing 'conventional LOH', the distance to the closest 'conventional LOH' was determined to be 0.

Normality of the distribution of delta values was tested using Blom's formula in order to ensure the correct model was being employed for power analysis. To establish differences in sensitivity and specificity of the CHC method with different contiguous point thresholds (CPT), correlations between CPT and the percentage of regions encompassing 'conventional LOH' or the distance of regions from 'conventional LOH' were derived using Pearsons correlation and the full data set for each CPT. In order to illustrate the fact that the primary determining factor for the distance of LOH regions inferred by CHC from the 'conventional LOH' was the resolution of the SNPs within the region, Pearsons correlation was used to compare the distance from the closest SNP showing 'conventional LOH' and the distance between SNPs. Only the 'inferred LOH' regions derived with a CPT of 5, and that did not encompass markers showing 'conventional LOH' (n = 159) were employed for this analysis. Demonstration of the need for the CHC method to have high heterozygosity frequencies at SNPs showing 'conventional LOH' in order to map LOH candidate regions over them when employing small cohort samples sizes was achieved by comparison of heterozygosity frequencies of SNPs showing 'conventional LOH' found inside candidate regions compared to those adjacent to candidate regions. This was achieved through the use of an independent-samples Students T-test of all regions generated with a CPT of 5. In order to highlight the utility of the enrichment score (ES) generated from the sum of delta values, Pearsons correlation was used to correlate the ES values of all 'inferred LOH' regions generated with a CPT of 5 with the distance of each region from the closest SNP showing 'conventional LOH'. To evaluate the efficacy of the HMM method, the percentage of HMM candidate regions that encompassed SNPs showing 'conventional LOH' was compared between patients by one-way ANOVA with Bonferroni post-hoc analysis.

### Cohort Heterozygosity Comparison Analysis of B-CLL, FL and DLBCL Samples

SNP microarray data for DLBCL, FL and B-CLL samples was analysed using the CHC method with reference to data generated from Caucasian HapMap samples. This data is freely available from http://www.affymetrix.com. It should be noted that different control samples were used for each NHL disease sub-category in order to avoid biasing the results. In order to ensure accuracy of LOH regions, CHC analysis was performed using a delta threshold of 0.4 and a CPT of 5. Regions of 'inferred LOH' were ranked by enrichment score and the top 2 regions for each subtype discussed.

## Implementation

### Selection of Informative Markers

Selection of informative SNPs (SNP_*i*_) is the first important aspect of the CHC method. As heterozygous genotypes are required to infer LOH using both the conventional and CHC methods of analysis, it is important that there be a likelihood of obtaining heterozygous genotypes within a case cohort. In order to perform cohort comparisons only those markers that were predicted to give a heterozygous call within the cohort were deemed to be informative; that is, only those markers with a population heterozygosity value (HV) greater than or equal to the reciprocal of the cohort size. HV for the given ethnicity is as determined by the SNP microarray annotation data. In the initial validation set of four samples in this investigation, 55% of the SNPs on the array were selected as informative markers; this translates to >125,000 SNPs. But by increasing the sample size in the secondary test sets of B-CLL, FL and DLBCL samples, larger numbers of the SNPs were selected as informative. Thus, the resolution of this method remains high even with low sample sizes, but is improved with increasing sample size due to inclusion of SNPs with lower heterozygosity frequencies.

### Calculation of Delta Values

Because of the potential to skew case and control heterozygosity values, all markers with absent genotype calls in any of the samples are removed from the analysis. Heterozygosity values are then calculated for all remaining SNPs for each cohort. A delta value (Δ), representative of the difference in heterozygosity value between each cohort, is then calculated for each SNP.

### Calculation of Delta Threshold

Different cohort sample sizes between investigations also make it important to calculate the delta threshold for each application of the CHC method. The statistical power of different investigations is dependant upon their respective sample sizes. With increasing sample size, a lesser effect size (Δ) is needed in order to obtain statistically significant results, whilst maintaining the desired minimum power (80%). The delta threshold is therefore set to maintain a statistical power of 80% (*α *= 0.05). For example, with the initial validation cohort (n = 4, *σ *= 0.3) the delta threshold was calculated to be 0.5, whereas by increasing the sample size to 10 in the secondary cohort of B-CLL samples while maintaining the same standard deviation the maximum required delta threshold required to obtain 80% power was lowered to 0.3. This means that with a sample size of 4 a 50% decrease in case cohort heterozygosity frequency must be observed in the case cohort compared to the reference cohort in order for the marker to be deemed informative, whereas with a sample size of 10 only a 30% decrease in case cohort heterozygosity frequency is required for a marker to be deemed informative. Increasing the cohort sample size when using the CHC method of LOH analysis is therefore expected to increase the specificity of the method and hence decrease the number of type I errors. Statistical power analysis was employed to calculate the delta value required (i.e. effect size) to achieve a minimum power of 80% with the specified cohort sample size (*α *= 0.05). The following formula was utilized to calculate the delta threshold (Δ_*T*_), which was used to determine which delta values were suggestive of LOH (Δ_*S*_). Within this formula, *σ *represents the standard deviation, Z represents the Z-score for a given value, a represents the level of significance (set to 0.05), *β *represents type II error rate and n represents the sample size.

### Contiguous Point Threshold and Inferring LOH

The number of contiguous SNPs required to infer LOH within a region is referred to as the contiguous point threshold (CPT). Strings of contiguous SNPs larger than or equal to the CPT and yielding informative delta values are highlighted as 'inferred LOH' regions. By altering this threshold users are able to modify the sensitivity and specificity of the CHC method. Using a low CPT a comparatively higher number of 'inferred LOH' regions can be localized compared to using a high CPT. However, this increased sensitivity is accompanied by decreased specificity. Setting the CPT is therefore a trade-off between sensitivity and specificity, and should be considered for each application of this method. However, it is recommended that a minimum CPT of 5 be employed in order to ensure sufficient specificity.

### Enrichment Score

In order to predict the proximity of 'inferred LOH' regions to markers showing 'conventional LOH', an enrichment score can be generated by summing the delta values generated within an 'inferred LOH' region. This enrichment score negatively correlates with the distance to closest 'conventional LOH' marker, indicating that the higher the enrichment score the closer the 'inferred LOH' regions will link to markers showing 'conventional LOH'. It is therefore recommended that the sequence immediately flanking CHC candidate regions with low enrichment scores also be considered when searching for candidate TSGs.

### Automated Analysis using Cohort Heterozygosity Comparison

A platform for automated CHC analysis (Additional file 1), instructions for use (Additional file 2), and the case and control input files (Additional files 3 and 4) utilized for analysis B-CLL, FL and DLBCL cohorts in this manuscript are available from the journal web-site.

## Results

### Validation of the Cohort Heterozygosity Comparison Method

Delta values were calculated for each informative SNP as described above. These values demonstrated a positively skewed normal distribution, as would be anticipated with decreased heterozygosity frequency in the case cohort. Calculation of the delta threshold is an important facet of the CHC method. With increasing cohort sample size a decrease in the effect size (Δ) is required to obtain significant results, whilst maintaining 80% power. For the initial validation set of B-CLL samples (n = 4, *σ *= 0.3) the delta threshold was calculated to be 0.5, for the subsequent test set (n = 10, *σ *= 0.3) the delta threshold was calculated to be 0.3.

The number of inferred regions for each CPT can be seen in Table 1. From this it can be seen that increasing the CPT decreased the number of LOH candidate regions generated via the CHC method. A significant positive correlation was found between CPT and the percentage of regions that spanned SNPs showing 'conventional LOH' (Pearsons correlation coefficient = 0.998; p = 0.002), and a significant negative correlation was found between CPT and the mean distance of 'inferred LOH' regions from the closest SNP showing 'conventional LOH' (Pearsons correlation coefficient = -0.996; p = 0.004). It is therefore predicted that raising the CPT increases the selectivity of the CHC method (i.e. decrease the chance of a Type I error), but in return may also decrease its sensitivity (i.e. increase the chance of a type II error).

**Table 1:** Validation of 'Inferred LOH' Regions Generated by the CHC Method

CPT	No. of Regions	Mean Region Size (bp)	Regions within 100 kb of LOH*	Mean Distance from LOH* (bp)
3	1,263	67,145	89%	36,460
4	594	101,166	92%	30,582
5	299	119,886	93%	25,220
6	162	112,431	96%	21,316

*'Conventional LOH'

A striking trend was also observed whereby those regions that did not encompass SNPs showing 'conventional LOH' mapped directly adjacent to them. When evaluating the LOH regions inferred with a CPT of 5, those SNPs showing 'conventional LOH' that mapped directly adjacent to regions of 'inferred LOH' were found to have a mean heterozygosity of 0.19 (n = 159). The mean population heterozygosity of SNPs showing 'conventional LOH' that mapped inside 'inferred LOH' regions was found to be 0.32 (n = 140), and was significantly higher than those mapping adjacent to candidate regions (p < 0.001). A positive correlation was found between the distance of candidate regions from the closest SNP showing 'conventional LOH' and the distance between SNPs (Pearsons correlation coefficient = 0.290; p = 0.022). A significant negative correlation was found between enrichment score and the distance to the closest SNP showing 'conventional LOH' (Pearsons correlation coefficient = -0.143; p < 0.001).

### Evaluation of the Hidden Markov Model Method

The HMM method for inferred LOH regions, associated with the Affymetrix GeneChip^® ^Genotyping Software CNAT 4.0 tool, generates scores of either 1 or 0 relating to 'inferred LOH' or retention of heterozygosity respectively. Regions with scores of 1 were classified as 'inferred LOH' regions and are summarized in Table 2. This method was found to generate large numbers of regions that spanned large genomic distances and that had a variable rate of success in encompassing 'conventional LOH' in the CLL patients. In total for the four cases, 699 regions were selected as regions of 'inferred LOH' by the HMM method. Of these, 82 regions overlapped in two patients, 22 candidate regions overlapped in three patients, and 2 candidate regions overlapped in all four patients, resulting in 567 independent regions of 'inferred LOH' for follow-up analysis from four cases. A significant difference in the percentage of HMM candidate regions encompassing SNPs showing 'conventional LOH' was also noted between cases (p = 0.01). No feature of individual case data was found to predict the percentage of regions encompassing SNPs showing 'conventional LOH'.

**Table 2:** Validation of 'Inferred LOH Regions Generated by the HMM Method

Sample	No. of Regions	Mean Region Size (bp)	Regions within 100 kb of LOH	Mean Distance from LOH (bp)
1	185	1,123,018	91%	33,850
2	187	1,003,721	78%	154,242
3	121	847,306	62%	230,310
4	206	892,220	97%	5,189
Total	699	975,359	82%	91,619

*'Conventional LOH'

### Cohort Heterozygosity Comparison Analysis of NHL Subtypes

**Figure 1:**
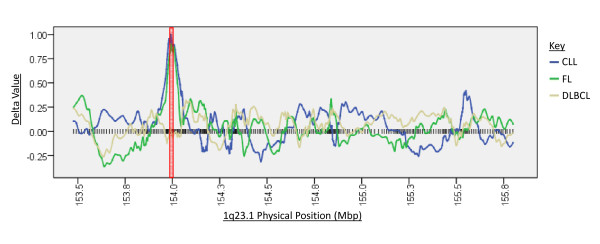
**Rolling window representation of delta values across the 1q23.1 cytoband for CLL, FL and DLBCL cohorts**. The position of each SNP over this region is shown by a black vertical bar. It can be seen that high delta values are obtained for contiguous SNPs over the ETV3 gene locus, marked with a red box, in CLL and FL cohorts but not in the DLBCL cohort.

After validation of the CHC method, SNP array data for DLBCL, FL and B-CLL samples was analysed using the CHC method. This generated 9 LOH candidate regions in DLBCL with enrichment scores ranging from 2.1 to 5.45, 10 LOH candidate regions in FL with enrichment scores ranging between 2.2 and 4.65, and 65 candidate regions in B-CLL with enrichment scores ranging from 2 to 5.8. The top two candidates for each NHL subtype, as assessed by enrichment score, are displayed on Table 3. Figure 1 shows a moving window analysis of the delta scores over the 1q32.1 candidate locus for all 3 of the NHL subtypes, showing its implication in CLL and FL but not DLBCL. The 1q32.1 region highlighted by the CHC method in CLL and FL cohorts, and the 3p25.3 region highlighted in the CLL cohort, was not detected by the HMM method in any of these samples. However, the HMM method did infer LOH over areas exceeding 500 Mbp in two FL samples that overlapped the comparatively smaller 5q11.2 candidate region highlighted by the CHC method. The HMM method also inferred LOH over a 965 Kbp in one DLBCL sample that overlapped the smaller 4q32.2 candidate region highlighted by the CHC method. Interestingly, the most notable overlap in candidate prediction between the HMM and CHC methods occurred at the 3q11.2 candidate region, in which the HMM region inferred LOH in 7 DLBCL samples with sizes ranging from 681 Kbp up to all of chromosome 3.

**Table 3:** Top 2 Regions of 'Inferred LOH' for each NHL Subtype

Disease	Location	Start Position	Finish Position	ES	Candidate
CLL	1q23.1	155269409	155393832	5.80	ETV3
CLL	3p25.3	9972054	10143892	5.80	FANCD2
FL	5q11.2	56022313	67005198	4.65	ERCC8
FL	1q23.1	155269409	155320805	3.55	ETV3
DLBCL	3q11.2	96553777	96717069	5.45	-
DLBCL	4q32.3	165412095	165440501	4.30	ANP32C

Top 'inferred LOH' regions, as determined by enrichment score, showing the cytogenetic location, start position and finish position determined by the location of first and last SNPs, and the candidate gene determined by annotation data.

## Discussion

We have developed a method for inferring regions of LOH by analyzing cohort heterozygosity values using SNP arrays, without the need for patient-matched samples. Initially, LOH regions were inferred in a small cohort of B-CLL patients using the Cohort Heterozygosity Comparison method. In order to investigate the efficacy of this method, regions of 'conventional LOH' were mapped in these patients through the use of SNP array data from patient-matched buccal samples, which was further validated by analysis of microsatellite markers. Following validation of this novel method of LOH analysis, CHC analysis was used to infer LOH in an extended cohort of B-CLL samples as well as larger cohorts of FL and DLBCL samples.

The CHC method employs genotype data generated by SNP microarray in order to detect regions of decreased heterozygosity frequency in the case cohort compared to the control cohort. Due to the decreasing heterozygosity of markers of markers on modern SNP array platforms, the CHC method only utilizes markers with a predicted capacity to generate at least one heterozygous genotype within each of the case and control cohorts. For each of these SNPs, delta values are calculated that correspond to the difference in heterozygosity between cases and controls. Decreases in heterozygosity frequency, as demonstrated by a positive delta score, are considered to be suggestive of LOH if they exceed a set threshold. This threshold (Δ_T_) is one of the modulatory parameters of the CHC method that can be used to alter the balance between sensitivity and specificity, but is usually set to maintain sufficient statistical power for the analysis. The second modulatory parameter contiguous point threshold (CPT), which is used to highlight regions as inferring LOH only if the number of contiguous markers exceeding Δ_T _is equal to or above the CPT. The CPT is applied in order to minimize false positives, and the ability of this parameter to modulate the sensitivity and specificity of this method is aptly shown by the decreasing number of inferred LOH regions and more accurate inference of LOH with increasing CPT, respectively (Table 1).

A caveat of the CHC method when employing small sample sizes is the increased requirement for high heterozygosity frequencies in order to detect LOH. This lead to a portion of the regions within the validation cohort mapping adjacent to, rather than encompassing, SNPs showing 'conventional LOH'. In this investigation the validation cohort sample size was modest; meaning that the effect size must be large in order to define regions with significantly decreased heterozygosity in the case cohort compared to the control cohort. In the validation cohort of 4 samples, when employing a CPT of 5 and a delta threshold of 0.5, there must be 5 consecutive SNPs with a 50% decrease in heterozygosity between the case and control cohorts in order LOH to be inferred. This means the CHC method can only detect LOH in small sample sizes if the region of LOH has a high enough initial rate of heterozygosity. In instances in which SNPs showing 'conventional LOH' had low population heterozygosity, the 'inferred LOH' region frequently mapped directly adjacent to them rather than encompassing them. This is supported by the fact that the SNPs showing 'conventional LOH' that mapped adjacent to regions of 'inferred LOH' derived by the CHC method had a significantly lower population heterozygosity than those that mapped inside the 'inferred LOH' regions. Furthermore, the distance of these adjacently positioned regions of 'inferred LOH' from 'conventional LOH' was determined primarily by the resolution of markers within each respective genomic region. The need for high population heterozygosity frequencies in order to map SNPs showing 'conventional LOH' within regions of 'inferred LOH' generated by the CHC method can be alleviated by increasing the cohort sample size. This would augment the power of the investigation and decrease the effect size required to define SNPs as being suggestive of LOH. However, even with the small sample size in the validation cohort the CHC method was shown to successfully infer LOH regions closely to SNPs showing 'conventional LOH', including those with low population heterozygosities. Furthermore, utilization of the CHC method with small sample sizes is further aided by the use of enrichment scores for each 'inferred LOH' region. This score is a sum of the heterozygosity frequency delta values, and was shown to significantly and negatively correlate with the distance of 'inferred LOH' from 'conventional LOH'.

The only current alternative to the CHC method for high resolution mapping of LOH using SNP microarray data without the need for patient-matched control samples is a method based on an HMM algorithm [7]. This method relies on identification of long strings of homozygous SNP genotypes in order to infer LOH. The HMM method therefore primarily differs from the CHC algorithm due to its mode of analysis being directed linearly within a single sample, while the CHC algorithm applies cross-sectional interrogation of genomic regions across entire cohorts of samples. This means that, while the HMM algorithm is predisposed to generating false-positive LOH calls as a result of consanguinity in individual patient's family trees and the subsequent enrichment of homozygosity, the cross-sectional approach adopted by the CHC algorithm is not as heavily affected by individual genetic background. We employed this method to analyze each of the four initial validation case samples in this investigation. It yielded 699 candidate regions with an average size of over 95 kb between the four cases. Only 19% of these candidate regions overlapped in two or more patients, resulting in 567 independent genomic regions that were suggestive of LOH. The HMM method does not provide a cumulative index or probability of LOH across all samples, and thus differences between this method and the CHC method are to be expected. However, when comparing the accuracy of the HMM-generated regions to the regions inferred by the CHC method with the recommended CPT, it can be seen that the CHC method delineated regions that were on average over 70% smaller, and more frequently mapped within the immediate vicinity of 'conventional LOH'. It should be noted that, although the size of HMM-inferred regions of LOH could be decreased by altering the state change parameter, this would also further decrease the specificity of the method. Nonetheless, the CHC method was found to infer LOH more specifically and within smaller genomic regions than the HMM method. This suggests that, not only is the CHC method more accurate in inferring LOH, but the increased precision may allow more specific delineation of smaller regions of LOH. Although there are clear advantages in combining cases into a cohort as part of the CHC method, this approach inhibits the ability to infer LOH regions within individual samples. Instead, the CHC method is more appropriately employed in generating candidate LOH regions implicating genes with a broad role in disease pathogenesis rather than a variable importance on a case-to-case basis.

In order to investigate whether automated analysis of larger samples of NHL patients could derive hypothetically important candidates, the CHC method was used to analyze larger cohorts B-CLL, FL and DLBCL samples. Using the enrichment score, the top two LOH candidate regions for each disease subtype were investigated for candidate genes that may possess a hypothetical tumor-suppressor function in NHL. Although one of the regions of 'inferred LOH' in DLBCL mapped over a genetic region with no closely linked coding loci, this region (3q11.2) has been shown to be lost in a range of cancers including acute lymphoblastic leukemia and mantle cell lymphoma [9,10], and the remaining 5 candidate regions were closely linked to attractive TSG candidates. Among these were two DNA repair genes (FANCD2, ERCC8), an inhibitor of RAS-mediated transformation (ANP32C), and a repressor of NFκB activity that was highlighted in both B-CLL and FL cohorts (ETV3).

The association between DNA repair genes and the pathogenesis of NHL is demonstrated by lymphoma predisposition within immunodeficiency cases containing mutations in genes mediating DNA damage repair [11] to developing NHL, as well as the numerous associations between polymorphisms in DNA repair genes and genetic susceptibility to NHL [12-14]. It is therefore not surprising that DNA repair genes may play a central tumor suppressor role in NHL. The FANCD2 gene was linked with a region of 'inferred LOH' highlighted in the B-CLL patient cohort, and functions in by forming complexes with BRCA1 or RAD51 and mediating repair of transcriptionally active genes [15]. Furthermore, this gene has also been shown to be essential for maintaining the G2 cell-cycle checkpoint [16]. The ERCC8 gene is also involved in DNA repair of transcriptionally active genes [17], and was linked with a region of inferred LOH in the FL patient cohort. Further support for this gene as a potential TSG is provided by the increased frequency of cancer in Cackayne's syndrome patients in which the ERCC8 gene is mutated [18]. The targeting of the FANCD2 and ERCC8 genes by LOH, as inferred by the CHC method, indicates that knock-out of their function may allow for the accumulation of mutations within transcriptionally active genes. Furthermore this may also be compounded by subsequent un-checked progression through the G2 checkpoint without FANCD2-associated cell-cycle arrest or apoptotic induction.

The ANP32C gene was highlighted as a potential TSG by CHC analysis of the DLBCL patient cohort. This gene has been previously described as a tumor suppressor gene, and elucidation of its function revealed that this role may be due to repression of RAS-mediated tumorigenesis [18]. However, our implication of this gene in the pathogenesis of NHL is a completely novel finding. The most interesting finding within the CHC analysis results was the revelation of ETV3 as a potential TSG in both B-CLL and FL patient cohorts. This gene is part of the ETS-family of tumor suppressors and functions in repression of NFκB-activated transcription [19]. LOH of the ETV3 locus at 1q23.1 has been previously demonstrated in B-CLL [20], and its potential as a TSG is supported by the importance of NFκB signaling in promoting proliferation and inhibiting apoptosis within lymphocytes [21]. The implication of genes with well defined tumor suppressor roles by cohort heterozygosity comparison of B-CLL, FL and DLBCL provides evidence that this novel method of analysis infers LOH within plausible TSGs. Although the implication of these candidate genes is only a preliminary finding, and validation by microsatellite analysis in a larger cohort of samples would be required in order to definitively link them with the pathogenesis of NHL, they clearly illustrate the utility of the CHC method for derivation of LOH candidates.

## Conclusions

We have developed a method of LOH analysis using case and unmatched control samples that differs from that which is currently available by directing the analysis cross-sectionally across multiple genomes within a cohort. Analysis of SNP array data with the CHC method was shown to infer LOH regions that linked closely to SNPs showing 'conventional LOH'. This method can be adopted for investigation of varied sample sizes, and the specificity and sensitivity of the method can be tailored to suit the objectives of each investigation. The candidate regions generated by the CHC method were considerably smaller and mapped significantly closer to SNPs showing 'conventional LOH' than the candidate regions generated by it's only current alternative, the HMM method. The utility of this method was further highlighted by its use to elucidate hypothetical, but potentially important, regions of LOH in three subtypes of NHL, including the ETV3 gene that was highlighted in both B-CLL and FL patient cohorts.

## Abbreviations

CHC: Cohort Heterozygosity Comparison; HMM: Hidden Markov Model; LOH: Loss of Heterozygosity; TSG: Tumor Suppressor Gene.

## Competing interests

The authors declare that they have no competing interests.

## Authors' contributions

MRG participated in DNA extractions, methological development and data analysis. RAL participated in methodological development. PJ participated in methodological development and software design. PW, JW and PM participated in patient recruitment and sample processing. LRG participated in methodological development and manuscript writing. All authors read and approved the final paper.

## Pre-publication history

The pre-publication history for this paper can be accessed here:

http://www.biomedcentral.com/1471-2407/10/195/prepub

## Supplementary Material

Additional file 1 **CHC Program**. Perl script for automated analysis using the cohort heterozygosity comparison method. Click here for file

Additional file 2 **Instructions for use of CHC Program**. Text File containing instructions for the use of the CHC Program. Click here for file

Additional file 3 **Example Case Data**. Text File containing genotype and annotation data for the extended B-CLL case cohort. Click here for file

Additional file 4 **Example Control Data**. Text File containing genotype and annotation data for the extended B-CLL control cohort. Click here for file

## References

[B1] Comings D A general theory of carcinogenesis Proc Natl Acad Sci 1971 70 3324 3328 10.1073/pnas.70.12.3324 PMC427229 4202843

[B2] Knudson A Two genetic hits (more or less) to cancer Nat Rev Cancer 2001 1 157 163 10.1038/35101031 11905807

[B3] Thiagalingam S Foy R Cheng K Lee H Thigalingam A Ponte J Loss of heterozygosity as a predictor to map tumor suppressor genes in cancer: molecular basis of its occurrence Curr Opin Oncol 2002 14 65 72 10.1097/00001622-200201000-00012 11790983

[B4] Lipshutz R Fodor S Gingeras T Lockhart D High density synthetic oligonucleotide arrays Nat Genet 1999 21 20 24 10.1038/4447 9915496

[B5] Collela S Yau C Taylor J Mirza G Butler H Clouston P Bassett A Seller A Holmes C Ragoussis J QuantiSNP: an objective Bayes Hidden-Markov Model to detect and accurately map copy number variation using SNP genotyping data Nuceic Acids Res 2007 35 2013 2015 10.1093/nar/gkm076 PMC1874617 17341461

[B6] Wang K Li M Hadley D Liu R Glessner J Grant S Hakonarson H Bucan M PennCNV: an integrated Hidden-Markov Model designed for high-resolution copy number variation detection in whole-genome SNP genotyping data Genome Res 17 1665 1674 10.1101/gr.6861907 17921354 PMC2045149

[B7] Beroukhim R Lin M Park Y Hao K Zhao X Garraway L Fox E Hochberg E Mellinghoff I Hofer M Descazeaud A Rubin M Meyerson M Wong W Sellers W Li C Inferring loss-of-heterozygosity from unpaired tumors using high-density oligonucleotide SNP arrays PLOS Comput Biol 2009 2 323 332 10.1371/journal.pcbi.0020041 PMC1458964 16699594

[B8] Lin M Wei L Sellers W Lieberfarb M Wong W Li C dChipSNP: significance curve and clustering of SNP-array-based loss-of-heterozygosity data Bioinformatics 2004 20 1233 1240 10.1093/bioinformatics/bth069 14871870

[B9] Tsuzuki S Karnan S Horibe K Matsumoto K Kato K Inukai T Goi K Sugita K Nakazawa S Kasugai Y Ueda R Seto M Genetic abnormalities involved in t(12;21) *TEL*-*AML1 *acute lymphoblastic leukemia: Analysis by means of array-based comparative genomic hybridization Cancer Sci 2007 98 698 706 10.1111/j.1349-7006.2007.00443.x 17374122 PMC11159317

[B10] Wlodarska I Pittaluga S Hagemeijer H De Wolf-Peeters C Berghe H Van Den Secondary chromosomal changes in mantle cell lymphoma Haematologica 1999 84 594 599 10406899

[B11] Tran H Nourse J Hall S Green M Griffiths L Gandhi M Immunodeficiency-associated lymphomas Blood Rev 2008 22 261 281 10.1016/j.blre.2008.03.009 18456377

[B12] Hill D Wang S Cerhan J Davis S Cozen W Severson R Hartge P Wacholder S Yeager M Chanock S Rothman N Risk of non-Hodgkin's lymphoma (NHL) in relation to germline variation in DNA repair and related genes Blood 2006 108 3161 3167 10.1182/blood-2005-01-026690 16857995 PMC1895525

[B13] Shen M Zheng T Lan Q Zhang Y Zahm S Wang S Holford T Leaderer B Yeager M Welch R Kang D Boyle P Zhang B Zou K Zhu Y Chanock S Rothman N Polymorphisms in DNA repair genes and risk of non- Hodgkin's lymphoma among women in Connecticut Hum Genet 2006 119 659 668 10.1007/s00439-006-0177-2 16738949

[B14] Ekstrom-Smedby K Lindgren C Hjalgrim H Humphreys K Schollkopf C Chang E Roos G Ryder L Falk K Palmgren J Kere J Melbye M Glimelius B Adami H Variation in DNA repair genes ERCC2, XRCC1, and XRCC3 and risk of Follicular Lymphoma Cancer Epidemiol Biomarkers Prev 2006 15 258 265 10.1158/1055-9965.EPI-05-0583 16492913

[B15] Taniguchi T Garcia-Higuera I Andreassen P Gregory R Grompe M D'Andrea A S-phase-specific interaction of the Fanconi anemia protein, FANCD2, with BRCA1 and RAD51 Blood 2002 100 2414 2420 10.1182/blood-2002-01-0278 12239151

[B16] Freie B Ciccone S Li X Plett P Orschell C Srour E Hanenberg H Shindler D Lee S Clapp W A role for the Fanconi anemia C protein in maintaining the DNA damage-induced G2 checkpoint J Biol Chem 2004 279 50986 50993 10.1074/jbc.M407160200 15377654

[B17] Hakem R DNA-damage repair; the good, the bad, and the ugly EMBO J 2008 27 589 605 10.1038/emboj.2008.15 18285820 PMC2262034

[B18] Bai J Brody J Kadkol S Pasternack G Tumor suppression and potentiation by manipulation of pp32 expression Oncogene 2001 20 2153 2160 10.1038/sj.onc.1204294 11360199

[B19] El Kasmi K Smith A Williams L Neale G Panopolous A Watowich S Haker H Foxwell B Murray P A transcriptional repressor and corepressor induced by the STAT3-regulated anti-inflammatory signaling pathway J Immunol 2007 179 7215 7219 18025162 10.4049/jimmunol.179.11.7215

[B20] Pfiefer D Pantic M Skatulla I Rawluk J Kreutz C Martenz U Fisch P Timmer J Veelken H Genome-wide analysis of DNA copy number and LOH in CLL using high-density SNP arrays Blood 2007 109 1202 1210 10.1182/blood-2006-07-034256 17053054

[B21] Baeuerle P Henkle T Function and activation of NFkappa-B in the immune system Ann Rev Immunol 1994 12 141 179 10.1146/annurev.iy.12.040194.001041 8011280

